# Risk factors of diarrhoeal disease in under-five children among health extension model and non-model families in Sheko district rural community, Southwest Ethiopia: comparative cross-sectional study

**DOI:** 10.1186/1471-2458-14-395

**Published:** 2014-04-23

**Authors:** Teklemichael Gebru, Mohammed Taha, Wondwosen Kassahun

**Affiliations:** 1 Department of Public Health, Aman Health Science College, Mizan Teferi, Ethiopia; 2 Department of Epidemiology, Jimma University, Jimma, Ethiopia; 3 Department of Biostatistics, Jimma University, Jimma, Ethiopia

**Keywords:** Risk factors, Under-five children, Health extension program, Model and non-model family

## Abstract

**Background:**

Worldwide diarrheal disease is the second leading cause of death in under-five year’s children. In Ethiopia diarrhoea kills half million under-five children every year second to pneumonia. Poor sanitation, unsafe water supply and inadequate personal hygiene are responsible for 90% of diarrhoea occurrence; these can be easily improved by health promotion and education. The Ethiopian government introduced a new initiative health extension programme in 2002/03 as a means of providing a comprehensive, universal, equitable and affordable health service. As a strategy of the programme; households have been graduated as model families after training and implementing the intervention packages. Therefore the aim of the study was to assess risk factor of diarrheal disease in under-five children among health extension model and non-model families.

**Method:**

A community based comparative cross-sectional study design was employed in 2012 at Sheko district. Multi-stage sampling technique was employed to select 275 model and 550 non-model households that had at least one under-five children. Data was collected using structured questioner and/or checklist by trained data collectors. A summery descriptive, binary and multivariate logistic regression was computed to describe the functional independent predictors of childhood diarrhoea.

**Result:**

The two weeks diarrhoea prevalence in under-five children among health extension model and non-model households were 6.4% and 25.5%, respectively. The independent predictors of childhood diarrhoea revealed in the study were being mothers can’t read and write [OR: 1.74, 95% CI: (1.03, 2.91)], monthly family income earn less than 650 Birr [OR: 1.75, 95% CI: (1.06, 2.88)], mothers hand washing not practice at critical time [OR: 2.21, 95% CI: (1.41, 3.46)], not soap use for hand washing [OR: 7.40, 95% CI: (2.61, 20.96)], improper refuse disposal [OR: 3.19, 95% CI: (1.89, 5.38)] and being non-model families for the health extension programme [OR: 4.50, 95% CI: (2.52, 8.03].

**Conclusion:**

The level of diarrheal disease variation was well explained by maternal education, income, personal hygiene, waste disposal system and the effect of health extension programme. Thus encouraging families to being model families for the programme and enhancing community based behavioural change communication that emphasize on personal hygiene and sanitation should be strengthening to reduce childhood diarrhoea.

## Background

Childhood mortality rates in general and infant mortality in particular, are often used as broad indicators of social development or as specific indicators of health status. Child mortality reduction by two-third is one target of Millennium Development Goal (MDG) [1]. Worldwide diarrheal disease is the second leading cause of death in under-five year children. It is responsible for 1.7 million morbidity and 760, 000 mortality of children every year [2]. In Ethiopia diarrhoea kills half million under-five children annually secondary to pneumonia. Poor sanitation, lack of access to clean water supply and inadequate personal hygiene are responsible for 90% of diarrheal disease occurrence, these can be easily improved by health promotion and education [3].

In effect, Ethiopia introduced a new initiative Health Extension program (HEP) in 2002/03 as a means of providing a comprehensive, universal, equitable and affordable health service for the rural population on the base of promotive, preventive and basic curative services. The programme was provided as a 16 packages focusing on health promotion and education supported by demonstration targeting households, particularly mothers and women through house to house visits [4].

As a strategy of this programme household have been graduated as model families; female and male household heads were selected and given basic training on the 16 health extension packages for 96 hours [5]. The graduated model families were expected to demonstrate practical changes in the use of health service program, environmental health, personal hygiene and serves as models to other community members. The strategy is based on the diffusion theory processed by which an innovation is communicated through certain channels over time among members of a social system [4].

However, there is no quantified evidence whether the health extension strategy has been made an effect on the risk factors of childhood diarrhoea. Therefore, the purpose of the study was to assess risk factor of diarrheal disease in under-five children among health extension model and non-model family in Sheko district rural community.

## Methods

A community based comparative cross-sectional study was conducted from January 31 to February 29/2012 in Sheko district. It is located in 577 km away from Addis Ababa to Southwest Ethiopia. The district has an estimated 57,397 total populations of these 8,960 are under five children and 11,714 households of these 2,390 are model families for health extension programme. The health service of the district is rendered through 3 health centers, 23 health posts and 7 different private owned clinics *[Sheko district health office: 2011 annual report]*. 

Source population of the study was all households that had at least one under-five children in the district rural community. Study population for model families were all households graduated (trained) by health extension, whereas all non-graduated households for non-model family that had at least one under-five children in randomly sampled Kebeles of the district.

Sample size was calculated using Epi-Info 7 statistical software by considering 25.1% prevalence of two weeks diarrhoea in under-five children for non-model family (as outcome variable) [6], 95% confidence interval (CI), 80% power, model to non-model household ratio 1:2, to detect 2 odds ratio (OR), design effect 2 and 90% response rate. Accordingly, the required total sample size was **825** (275 model and 550 non-model) households that had at least one under five-children were included in the study.

A multi-stage sampling procedure was employed to select study participants. First eleven Kebeles from the twenty three Kebeles' were selected randomly using lottery method as primary sampling unit (PSU). After sampling frame preparation, the calculated sample sizes were allocated proportional to size for each selected Kebele. Then, simple random sampling was applied to select households that had at least one under-five children as secondary sampling unit (SSU).

An adapted WHO core questioner and checklist were used to collect the data by eleven trained diploma holder data collectors using interview and observation for environmental and water supply factors [7,8]. The instrument contains socioeconomic, environmental, water supply and behavioural conditions.

To enhance instrument reliability, the adapted questioner was translated in to the local language Amharic and back translated in to English by another person. Moreover, the instrument was pre-tested on 5% of the actual sample size and necessary corrections were made accordingly. A three-day training was given to data collectors and supervisors prior the data collection. The data collection process was checked on daily base by three BSc holder supervisors and principal investigator. Then double data entry was made using Epi-data 3.1 software.

After data processing, analysis was made using SPSS version 20.0. A summary descriptive statistics was computed. Variables in binary screening found at p-value ≤ 0.25 were further considered into multiple logistic regression to avoid unstable estimate [9]. Finally backward stepwise logistic regression analysis was applied to describe the functional independent predictors of childhood diarrhoea. A point estimates of Odds ratio (OR) with 95% confidence interval (CI) were determined to assess the strength of association between independent and dependent variable. For all statistical significant tests p-value < 0.05 was used as a cut-off point.

The study was ethically approved by the health research and post graduate coordinating office, college of Public health and medical science of Jimma University. Oral consent was obtained from each study participants before each interview and confidentiality was assured.

### Operational definitions

1) ***Model family*:** household head/caregiver, which had taken basic training for 96 hours and graduated on the 16 health extension packages.

2) ***Non-model family*:** household head/caregiver, which had not taken basic training on the 16 health extension packages.

3) ***Kebele*:** the lowest administrative unit in Ethiopia, which resides 500 households.

4) ***Hand washing at critical time*:** if a mother/caregiver practiced all simple hand washings before food preparation, before child feeding, after child cleaning and after latrine visiting was considered as “all practiced” unless considered as “partially practiced”.

5) ***Proper refuse disposal***: is a way of disposal refuses which includes burning, buried in pit or store in a container, compost, and disposed in designed site, whereas disposing in open field was considered as improper refuse disposal.

## Result

A total of 825 (275 model and 550 non-model) households that had at least one under-five children were planned to participate in the study, out of these 794 (265 model and 529 non-model) were enrolled in the data collection, which makes a response rate of 96.2%.

### Socio demographic characteristics

In this study almost all of the respondents were real mothers of the index child for both groups [264 (99.6%) model and 528 (99.8%) non-model households]. Regarding to religion Orthodox was shared more than half of the total study population for both group [167 (63.0%), model and 332 (62.8%), non-model households]. Majority of the mothers 260 (98.1%) model and 515 (97.4%) non-model mothers occupation were housewife and almost three fourth of the study population were can’t read and write by both groups [194 (73.2%) model and 400 (75.6%) non-model households].

Among the interviewed households 175 (66.0%) model and 382 (72.2%) non-model their family size were less than or equal to five. During the data collection time majority of the households 204 (77.0%) model and 437 (82.6%) non-model were earn monthly income less than or equal to 650.00 Ethiopian Birr.

### Environmental conditions

Almost all households of model and non-model families included in this survey had have owned private latrine [265 (100%) model, and 516 (97.5%) non-model], but out of those latrine owner 93 (35.1%) model and 307 (59.5%) non-model households were in need of their latrine being maintenance. Among the total households interviewed in this survey 215 households had have well-water in their compound. Well-water distance less than 15 meter from latrine accounts almost similar by both groups [185 (69.8%) model and 322 (62.4%) non-model households]. Hand washing facility near to latrine was more common among model households 210 (79.2%) than non-model households111 (21.5%). Proper refuse disposal was also more practiced by model households 249 (93.9%) as compared to non-model households 351 (66.3%) (Table 1).

**Table 1:** Possible environmental factors associated with childhood diarrhoea (n = 794), Sheko district rural community, Southwest Ethiopia, January – February 2012

**Variables**	**Model family**		**Non-model family**	
	***Diarrhoea***		***Diarrhoea***	
	**Yes**	**No**	**Yes**	**No**
	**N (%)**	**N (%)**	**N (%)**	**N (%)**
**Climatic zone**				
“Kolla”	12 (4.5)	156 (58.9)	89 (16.8)	245 (46.3)
“Woina dega”	4 (1.5)	56 (21.1)	29 (5.5)	90 (17.0)
“Dega”	1 (0.4)	36 (13.6)	17 (3.2)	59 (11.2)
**Latrine availability**				
Yes	17 (6.4)	248 (93.6)	127 (24.0)	389 (73.5)
No	0 (0.0)	0 (0.0)	8 (1.5)	5 (0.9)
**latrine maintenance**				
No need	1 (0.4)	171 (64.5)	75 (14.5)	134 (26.0)
Needed	16 (6.0)	77 (29.1	52 (10.1)	255 (49.4)
**Latrine location from well water**				
Uphill	7 (8.2)	23 (27.1)	33 (25.4)	22 (16.9)
Same level	4 (4.7)	6 (7.1)	21 (16.2)	4 (3.1)
Downward	3 (3.5)	42 (49.4)	21 (16.2)	29 (22.3)
**Latrine distance from well water**				
< 15 meters	5 (5.9)	14 (16.5)	53 (40.8)	14 (10.8)
15–30 meters	3 (3.5)	18 (21.2)	7 (5.4)	23 (17.7)
> 30 meters	6 (7.1)	39 (45.9)	15 (11.5)	18 (13.8)
**Hand washing facility near to latrine**				
Yes	13 (4.9)	197 (74.3)	30 (5.8)	81 (15.7)
No	4 (1.5)	51 (19.2)	97 (18.8)	308 (59.7)
**House shared with domestic animals**				
Yes	4 (1.5)	56 (21.1)	38 (7.2)	142 (26.6)
No	13 (4.9)	192 (72.5)	97 (18.3)	252 (47.6)
**Refuse disposal method**				
Proper disposal	17 (6.4)	232 (87.5)	106 (20.0)	245 (46.3)
Improper disposal	0 (0.0)	16 (6.0)	29 (5.5)	149 (28.2)

### Water supply related factors

Protected water source selection was more preferred by model households 165 (62.3%) than non-model households 288 (54.4%). Water treatment practice at home level was also more practiced among model households 151 (57.3%) than only 87 (16.5%) non-model households. Even though half of both model and non-model households can be accessible for water within 15 minutes [134 (50.6%) model and 265 (50.1%) non-model households], but majority of their water consumption was less than 7.5 litters per person per day by both model and non-model households [165 Z (62.2%) model and 332 (63.7%) non-model households] (Table 2).

**Table 2:** Possible water supply conditions associated with childhood diarrhoea (n = 794), Sheko district rural community, Southwest Ethiopia, January – February 2012

**Variables**	**Model family**		**Non-model family**	
	***Diarrhoea***		***Diarrhoea***	
	**Yes**	**No**	**Yes**	**No**
	**N (%)**	**N (%)**	**N (%)**	**N (%)**
**Main water source**				
Unprotected	7 (2.6)	93 (35.1)	80 (15.1)	161 (30.4)
Protected	10 (3.8)	155 (58.5)	55 (10.4)	233 (44.0)
**Time to fetch water (minutes)**				
< 15	4 (1.5)	130 (49.1)	86 (16.3)	179 (33.8)
15-30	6 (2.3)	62 (23.4)	34 (6.4)	137 (25.9)
> 30	7 (2.6)	56 (21.1)	15 (2.8)	78 (14.7)
**Water container cleanness**				
Yes	17 (6.4)	247 (93.2)	122 (23.1)	340 (64.3)
No	0 (0.0)	1 (0.4)	13 (2.5)	54 (10.2)
water consumption (L/p/d^*^)				
≤ 7.5 litters	8 (3.0)	157 (59.2)	89 (16.8)	243 (45.9)
> 7.5 litters	9 (3.4)	91 (34.3)	46 (8.7)	151 (28.5)
**Water treatment at home**				
Yes	8 (3.0)	144 (54.3)	22 (4.2)	65 (12.3)
No	9 (3.4)	104 (39.2)	113 (21.4)	329 (62.2)

^*^L/p/d: Litters/person/day.

### Behavioural conditions

Proper latrine utilization was more practiced by model households 265 (100%) than non-model households 429 (83.1%). Majority of both groups used latrine always [261 (98.1%) model and 478 (92.7%) non-model]. Proper children’s stool disposal was more practiced by model households 247 (93.2%) than non-model households 307 (58.1%).

Hand washing at critical time were more exercised by model respondents 163 (61.5%) than non-model respondents 195 (36.9%). Soap utilization for hand washing was also more practiced 91 (34.3%) by model respondents than 50 (9.5%) non model respondents (Table 3).

**Table 3:** Possible behavioural factors associated with childhood diarrhoea (n = 794), Sheko district rural community, Southwest Ethiopia, January – February 2012

**Variables**	**Model family**		**Non-model family**	
	***Diarrhoea***		***Diarrhoea***	
	**Yes**	**No**	**Yes**	**No**
	**N (%)**	**N (%)**	**N (%)**	**N (%)**
**Proper latrine utilization**				
Yes	17 (6.4)	248 (93.6)	108 (20.9)	321 (62.2)
No	0 (0.0)	0 (0.0)	19 (3.7)	68 (13.2)
**Latrine utilization condition**				
Always	17 (6.4)	244 (92.1)	119 (23.1)	359 (69.6)
Mostly	0 (0.0)	3 (1.1)	5 (1.0)	27 (5.2)
Rarely	0 (0.0)	1 (0.4)	3 (0.6)	3 (0.6)
**Children's stool disposing method**				
Proper	15 (5.7)	232 (87.5)	86 (16.3)	221 (41.8)
Improper	2 (0.8)	16 (6.0)	49 (9.3)	173 (32.7)
**Hand washing at critical time**				
All practiced	3 (1.1)	160 (60.4)	40 (7.6)	155 (29.3)
Partially practiced	14 (5.3)	88 (33.2)	95 (18.0)	239 (45.2)
**Soap utilization for hand washing**				
Yes	0 (0.0)	91 (34.33)	4 (0.76)	46 (8.70)
No	17 (6.42)	157 (59.24)	131 (24.76)	348 (65.78)

### Diarrhoea prevalence

Comparing model and non-model households, a remarkable difference in childhood diarrhoea prevalence was observed. The occurrence of diarrheal disease among children’s whose families were non-model for health extension program was 25.5%, which is much more common than children’s whose families were model for the program was 6.4%.

### Independent factors associated with childhood diarrhoea

Independent factors were assessed by constructing selective model based on bivariate screening used as a first step to avoid an excessive number of variables and unstable estimates. Variables in the bivariate analysis of socio-economic, environmental conditions, behavioural conditions and child characteristics with respect to childhood diarrhoea; which were found at p-value ≤ 0.25 were further considered in to final model backward stepwise multiple logistic regression analysis. Accordingly, being mothers can’t read and write, monthly family income earn ≤ 650.00 Birr, poor hand washing practice at critical time and soap use, improper refuse disposal method, and being non-mode household for health extension programme were found independent predictor for the occurrence of childhood diarrhoea (Table 4).

**Table 4:** Independent factors associated with childhood diarrhoea (n = 794), Sheko district rural community, Southwest Ethiopia, January – February 2012

**Variables**	**Childhood diarrhoea**	
	**OR (with 95% CI)**	
	***Crude***	***Adjusted***
**Mother education**		
Literate	1.00	1.00
Can’t read & write	1.62 (1.03, 2.53)	**1.74 (1.03, 2.91)**
**Monthly family income**		
> 650.00 Birr	1.00	1.00
< = 650.00 Birr	1.32 (0.86, 2.03)	**1.75 (1.06, 2.88)**
**Hand washing at critical time**		
All practiced	1.00	1.00
Partially practiced	2.44 (1.66, 3.59)	**2.21 (1.41, 3.46)**
**Soap use for hand washing**		
Yes	1.00	1.00
No	10.03 (3.65, 27.58)	**7.40 (2.61, 20.96)**
**Refuse disposal method**		
Proper	1.00	1.00
Improper	2.22 (1.40, 3.51)	**3.19 (1.89, 5.38)**
**Household condition for health extension**		
Model family	1.00	1.00
Non-model family	4.99 (2.94, 8.48)	**4.50 (2.52, 8.03)**

Children’s whose mothers cannot read and write were 1.74 times more likely to concede diarrhoea than children’s whose mothers were literate [OR: 1.74, 95% CI: (1.03, 2.91)]. Children’s whose families monthly income earn less than or equal to 650 Birr were 1.75 times more likely to concede diarrhoea than children’s whose families monthly income were greater than 650 Birr [OR: 1.75, 95% CI: (1.06, 2.88)].

Children’s whose mother didn’t practiced hand washing at critical time were 2.21 times more likely to concede diarrhoea than children’s whose mothers were practiced hand washing at critical time [OR: 2.21, 95% CI: (1.41, 3.46)]. Children’s whose mothers didn’t used soap for hand washing were 7.40 times more likely to concede diarrhoea than children’s whose mothers were used soap for hand washing [OR: 7.40, 95% CI: (2.61, 20.96)].

Children’s whose families practiced improper refuse disposal were 3.19 times more likely to concede diarrhoea than children’s whose families were practiced proper refuse disposal [OR: 3.19, 95% CI: (1.89, 5.38)].

Children’s whose families were non-model for the health extension programme were 4.50 times more likely to have diarrhoea than children’s whose families were model for the health extension programme [OR: 4.50, 95% CI: (2.52, 8.03)].

## Discussion

Children whose mothers can’t read and write were more likely to have diarrhoea when compared with children whose mothers were literate. This finding was similar with other studies, where the prevalence of diarrhoea varies according to education of mothers which was relatively high among children whose mother don’t read and write [10,11]. Since education provides the knowledge on the rules of hygiene, feeding and weaning practices [12,13].

Children whose families earn monthly income less than or equal to 650 Ethiopian Birr were more likely to develop diarrhoea when compared with children whose families income were greater than 650 Ethiopian Birr. This result was consistent with other studies, where mostly diarrhoea often linked with hygiene, water and sanitation. The rich families have used soap for hand washing, aqua-guard at their houses to protect bacterial contamination in water and also constructed toilets, however low income families suffering from this disease because they can’t afford these things [14].

In this study there were a remarkable difference of childhood diarrhoea were observed among children whose mothers not practiced hand were washing at critical time with soap were more likely to develop diarrhoea when compared to children whose mothers were practiced hand washing at critical time with soap. This was consistent with study finding, where mothers are the main caregivers for their children they may have prior knowledge acquired from health extension programme, their experience and/or formal education [15-17]. Consequently, Mothers probably wash their hand in order to prevent diarrhoea and occurrence of other hygiene related communicable disease.

Children’s whose families practiced improper refuse disposal were more likely to develop diarrhoea when compared to children whose families were practiced proper refuse disposal. This result was consistent with other reports, where environmental sanitations most often linked with the diarrhoea is refuse disposal. Poor refuse disposal is attributed to direct contact with human excreta when the child starts to crawl, and easily accessible for vector and rodents, which are means of diarrhoea transmission so refuse disposal had important role in diarrhoea in the study area [16,18,19].

Children whose family’s non-model for health extension programme were more likely to develop diarrhoea when compared to children whose families were model for health extension programme. Health promotion and education supported by demonstration on personal hygiene, water supply safety measure and waste management are important to prevent diarrhoea. Model family has created synergy on these things for their better health, however non-model families suffering from diarrheal disease, which was particularly pervasive in the conditions of poor personal hygiene and poor sanitation practice [20].

In this study the climatic zone, sanitary facility (latrine and hand washing) and water supply (source, distance and home based treatment) were not associated with the occurrence of diarrhoea, which contrasts with previous studies [12,21,22]. This might be due to homogenous effect.

The two weeks diarrhoea prevalence in under-five year of age children whose family’s non-model for health extension was more prone than children whose family’s model for the programme. This result was consistent with other report, where sanitation hardware interventions were as effective as hygiene software and water quality, leading to a 37% relative reduction in diarrhea morbidity [23].

The validity of the study may be limited by a cross-sectional rather than longitudinal design of the study and information contamination. However, this is the first study in the area and we believe, it raises awareness in Ethiopia that will add valuable information to the existing healthcare service.

## Conclusion

In conclusion, the variation in the level of diarrheal morbidity was well explained by maternal education, income, personal hygiene, refuse disposal system and the effect of health extension programme. Cognizant of this fact, we recommend that, the strategy of being model families and, behavioural change communication education emphasized on personal hygiene lead to total sanitation should be strengthen to reduce the risk of childhood diarrhoea.

## Competing interests

The authors declare that they have no competing interests.

## Authors’ contributions

TG conceived and designed the study, and analysed the data. MT, WK contributed to the design of the study. All authors contributed to the manuscript and approved its final version submitted for publication.

## Pre-publication history

The pre-publication history for this paper can be accessed here:

http://www.biomedcentral.com/1471-2458/14/395/prepub
